# Machine learning-based preoperative prediction of perioperative venous thromboembolism in Chinese lung cancer patients: a retrospective cohort study

**DOI:** 10.3389/fonc.2025.1588817

**Published:** 2025-06-25

**Authors:** Zhe Chen, Min Qiang, Yang Hong, Weibo Tian, Mingbo Tang, Wei Liu

**Affiliations:** ^1^ Department of Thoracic Surgery, The First Hospital of Jilin University, Changchun, China; ^2^ College of Clinical Medicine, Jilin University, Changchun, China; ^3^ Vall d'Hebron Institute of Research (VHIR), Barcelona, Spain; ^4^ Department of Neurology, The First Hospital of Jilin University, Changchun, China

**Keywords:** lung cancer, perioperative period, venous thromboembolism, machine learning, prediction model

## Abstract

**Background:**

Perioperative venous thromboembolism (VTE) is a severe complication in lung cancer surgery. Traditional prediction models have limitations in handling complex clinical data, whereas machine learning (ML) offers enhanced predictive accuracy. This study aimed to develop and validate an ML-based model for preoperative VTE risk assessment.

**Methods:**

A retrospective cohort of 1,013 lung cancer patients who underwent surgery at the First Hospital of Jilin University (April 2021–December 2023) was analyzed. Preoperative clinical and laboratory data were collected, and six key predictors—age, mean corpuscular volume, mean corpuscular hemoglobin, fibrinogen, D-dimer, and albumin—were identified using univariate analysis and Lasso regression. Eight ML models, including extreme gradient boosting (XGB), random forest, logistic regression, and support vector machines, were trained and evaluated using AUC, precision-recall curves, decision curve analysis, and calibration curves.

**Results:**

VTE occurred in 175 patients (17.3%). The XGB model demonstrated the highest predictive performance (AUC: 0.99 training, 0.66 validation; AUPRC: 0.323), with age and mean corpuscular volume identified as the most influential predictors. An online prediction tool was developed for clinical application.

**Conclusion:**

The ML-based XGB model provides a reliable preoperative risk assessment for VTE in lung cancer patients, enabling early risk stratification and personalized thromboprophylaxis.

## 1 Introduction

Lung cancer remains a leading cause of cancer-related mortality worldwide, accounting for a substantial proportion of global cancer deaths (1). Surgical intervention plays a crucial role in the treatment of lung cancer, particularly in the early stages of the disease, where it not only has the potential to cure the disease but also significantly improves patient survival rates (2). However, perioperative venous thromboembolism (VTE), including deep vein thrombosis (DVT) and pulmonary embolism (PE), remains one of the most serious complications after surgery, significantly affecting patient morbidity and mortality (3). Studies have shown that the incidence of VTE in lung cancer patients can be as high as 13.2% in the first year after surgery (4). During this period, the combination of surgical stress and the hypercoagulable state associated with malignancy significantly increases the risk of thrombotic events, leading to severe complications, prolonged hospital stays, and increased mortality (5). Therefore, how to effectively identify high-risk patients before surgery and take timely, targeted interventions has become an urgent clinical challenge.

Traditional risk assessment models, such as multivariable logistic regression analysis, have played an important role in identifying risk factors for VTE (6). These models provide valuable insights into the relationships between various clinical variables and the occurrence of VTE. However, as the complexity of clinical data increases and the demand for more precise predictive models rises, the limitations of traditional statistical methods have become increasingly apparent. Therefore, there is an urgent need for new reliable methods to predict perioperative thrombosis.

Machine learning (ML) is an innovative, computer-based approach that has been widely applied in the field of medical data analysis in recent years (7, 8). The core principle is to extract patterns from data and generate predictions (9). Supervised learning is one of the main methods, where models are trained using fully labeled data to improve prediction accuracy. Unlike traditional statistical methods, such as logistic regression, machine learning does not rely on predefined models. By iteratively refining algorithms, it can more effectively identify complex interactions between variables (10).

In the past few years, machine learning (ML) algorithms, including logistic regression (LR), decision trees (DT), random forest (RF), naive bayes (NB), gradient boosting(GB), support vector machines (SVM), and k-nearest neighbors (KNN), have been increasingly applied to risk prediction in lung cancer (11) and other cancers (12, 13) and cancer-related complications (14, 15), significantly improving the accuracy of risk assessment. These findings indicate that machine learning holds great promise for disease risk prediction, especially in uncovering complex patterns in multidimensional data.

This study aims to combine traditional statistical methods with advanced machine learning techniques to establish a preoperative predictive model for perioperative VTE in lung cancer patients. By incorporating preoperative indicators, we will select the optimal predictive model based on the area under the receiver operating characteristic curve (AUC), area under the precision-recall curve (AUPRC), decision curve analysis (DCA), and calibration curve (CC). Furthermore, we will rank the preoperative predictors in order of importance and identify the most important predictors. The findings of this study may significantly enhance clinical decision-making and provide guidance for the development of preoperative VTE prevention strategies for lung cancer surgical patients.

## 2 Materials and methods

### 2.1 Data collection

#### 2.1.1 Study subjects

This study included 1,013 lung cancer patients who underwent surgery at the First Hospital of Jilin University, Thoracic Surgery Department, between April 2021 and December 2023. All patients were diagnosed with primary malignant lung tumors postoperatively. Clinical data, including perioperative bilateral lower limb ultrasound examinations, were collected through the electronic medical record system.

All patients underwent bedside bilateral lower limb color Doppler ultrasonography before and after surgery to detect the presence or absence of deep vein thrombosis (DVT). Examinations were performed by trained vascular sonographers using standardized protocols, and diagnostic criteria followed international guidelines for compressibility and intraluminal filling defects.

##### 2.1.1.1 Inclusion criteria

1. Underwent surgery at our hospital;
2. Postoperative pathological diagnosis confirmed primary malignant lung tumor;
3. Preoperative bilateral venous color ultrasound examination showed no thrombosis, and postoperative color ultrasound was performed;
4. Patients were able to cooperate with medical history inquiries and routine examinations, and clinical data were complete.

##### 2.1.1.2 Exclusion criteria

1. Postoperative diagnosis revealed benign lung disease, secondary malignant tumor, or indeterminate pathological staging;
2. Preoperative or postoperative venous ultrasound of the lower limbs was not performed, or thrombosis was detected preoperatively;
3. Patients with hematological diseases (e.g., hemophilia, thrombocytopenia);
4. Use of anticoagulant drugs preoperatively;
5. History of VTE.

#### 2.1.2 Clinical data collection

The data collection was classified into three primary categories: general condition, preoperative laboratory assessments, and tumor characteristics. A total of 43 variables were meticulously collected, encompassing patient demographic information, preoperative laboratory findings, and detailed pathological and imaging characteristics of the tumor.

### 2.2 Data preprocessing

Missing values were handled differently for numerical and categorical features. For numerical variables, missing data were imputed iteratively using the Bayesian ridge regression estimator. In contrast, categorical variables were treated as additional categories to account for missing values. Subsequently, one-hot encoding was applied to categorical variables such as “gender” to generate binary representations. Numerical variables were standardized to have a mean of zero and a variance of one. Continuous variables were summarized using their count, mean, and standard deviation, while categorical variables were described by their count.

### 2.3 Feature selection

Statistical analysis was conducted using IBM SPSS Statistics 29. The Kolmogorov-Smirnov test was used to assess data normality. For normally distributed continuous variables, data were presented as mean ± standard deviation (Mean ± SD), and independent sample t-tests were used to compare group differences. For non-normally distributed data, medians and interquartile ranges were used, with Mann-Whitney U tests applied for comparison. Categorical variables were expressed as percentages (%), and group differences were compared using the chi-square test (χ²). Statistical significance was set at α = 0.05, with P < 0.05 indicating a significant difference.

Lasso regression was conducted using R version 4.3.1. All variables were standardized, and the `cv.glmnet` function was used for cross-validation to select the optimal regularization parameter lambda. Lasso regression applied L1 regularization to select important variables. The model’s fit was evaluated using the mean squared error (MSE). All tests were set with a significance level of α = 0.05, with P < 0.05 indicating statistical significance.

Variables that showed statistical significance in both univariate analysis and Lasso regression were included in the model.

### 2.4 Learning algorithms

The subjects were randomly divided into a training set (n = 710) and a test set (n = 303) at a 7:3 ratio. To mitigate class imbalance, the Synthetic Minority Oversampling Technique (SMOTE) was applied during the training phase. We evaluated the performance of various models, including extreme gradient boosting machine (XGB), random forest (RF), decision tree (DT), K-nearest neighbors (KNN), multilayer perceptron (MLP), logistic regression (LR), support vector machine (SVM), and Naive Bayes (NB), selecting the best-performing classifier for prediction. Hyperparameter optimization was performed using Bayesian optimization on the validation set to prevent overfitting. A 10-fold cross-validation was applied to each hyperparameter set, with the validation set used for performance assessment. Eight models were trained on the training set data. For hyperparameter tuning, a random search was conducted, with 80% of the dataset allocated for model fitting and 20% for validation. The resulting models were subsequently validated and assessed using validation data. The final network predictor was developed based on the model that exhibited the best performance among the eight evaluated. The code for the data analysis is provided in the supporting materials.

Feature importance was assessed during training through permutation importance and impurity-based scores. Impurity-based scores reflect the contribution of each feature by evaluating its frequency as a decision node and its impact on entropy reduction. Combined with permutation importance, which measures accuracy loss from feature shuffling, we derived a robust feature ranking.

### 2.5 Method evaluation

To assess the performance of the ML models, we calculated and compared the area under the receiver operating characteristic curve (AUROC). AUROC represents the probability that the model ranks a randomly chosen VTE patient higher than a randomly chosen non-VTE patient, with a higher AUROC indicating better performance. However, AUROC can sometimes be misleading, particularly in imbalanced datasets, where it may overestimate performance compared to the area under the precision-recall curve (AUPRC). To address this, we also calculated AUPRC (or Average Precision) to account for AUROC’s limitations. Average Precision is derived by summing the precision at each threshold, weighted by the increase in recall from the previous threshold. AUPRC provides a better understanding of the model’s ability to correctly identify VTE patients while minimizing false positives.

In addition to AUROC, we incorporated decision curve analysis (DCA) to evaluate the model’s clinical utility in real-world decision-making. DCA calculates the net benefit at different thresholds by balancing the correct identification of positive cases with the cost of false positives. Unlike AUROC, which focuses solely on classification performance, DCA considers the practical impact of the model’s predictions across various clinical scenarios.

To further evaluate the performance of the ML models, we also computed and analyzed the calibration curve (CC). The calibration curve assesses the agreement between predicted probabilities and actual outcomes, providing insight into how well the model’s predicted probabilities align with real-world probabilities. Unlike AUROC and AUPRC, which focus on classification accuracy and precision, the calibration curve emphasizes the model’s reliability in estimating the likelihood of an event. This metric is essential in determining how trustworthy the model’s predictions are for clinical decision-making, ensuring that the predicted probabilities are not biased or overestimated.

## 3 Results

### 3.1 Characteristics of the study cohort

Our analysis included a total of 1,013 patients, of whom 175 experienced VTE (venous thromboembolism). Our data indicate that patients with venous thromboembolism (VTE) exhibit significant differences in several clinical characteristics compared to those without VTE. Patients with VTE are older and have a slightly lower proportion of males. Notable biochemical markers include higher preoperative D-dimer levels and lower albumin levels in the VTE group. Additionally, statistically significant differences were observed between the groups in preoperative mean corpuscular volume (MCV), hemoglobin levels, and Cyfra 21–1 values. The details of all these clinical characteristics are shown in **Table 1**.

**Table 1:** Clinical Characteristics of Patients with and without Lower Limb VTE.

Variable	Category	VTE Group (n=175)	Non-VTE Group (n=838))	P-value
Gender	Male Female	54(31.4%) 121(68.6%)	301(35.7%) 537(64.3%)	P=0.04
Age (years)		63.02±7.21	56.69±10.40	P<0.01
Height (cm) Weight (kg)		163.41±6.12 62.84±9.83	164.22±7.21 64.49±10.98	P=0.19 P=0.09
BMI		23.50±3.24	23.83±3.17	P=0.24
Hypertension	Yes No	36(19.8%) 139(80.2%)	172(16.7%) 666(83.3%)	P=0.64
Diabetes	Yes No	15(8.1%) 160(91.9%)	99(11.8%) 739(88.1%)	P=0.25
Smoking	Yes No	44(24.4%) 131(75.6%)	150(17.9%) 688(82.1%)	P=0.27
Alcohol Consumption	Yes No	19(9.3%) 156(90.7%)	70(7.2%) 768(92.8%)	P=0.46
Stroke	Yes No	9(3.5%) 166(96.5%)	24(2.4%) 814(97.6%)	P=0.62
FEV1/FVC%		87.31±5.20	87.57±6.96	P=0.82
Ejection Fraction (EF%)		64.11±3.39	64.62±3.76	P=0.26
ESR (mm/h)		12.20±8.16	13.31±12.40	P=0.42
Blood Type	A B AB O	41(23.3%) 69(39.5%) 30(17.4%) 35(19.8%)	223(26.8%) 265(31.6%) 127(15.0%) 223(26.6%)	P=0.67
Preoperative WBC count(10^^9^/L)		5.90±1.96	5.78±1.62	P=0.67
Preoperative Neutrophil		0.55±0.09	0.55±0.09	P=0.8
Preoperative Lymphocyte		0.35±0.09	0.35±0.08	P=0.84
Preoperative Monocyte		0.07±0.02	0.07±0.02	P=0.94
Preoperative Neutrophil count(10^^9^/L)		3.59±2.70	3.23±1.21	P=0.08
Preoperative Lymphocyte count(10^^9^/L)		1.96±0.62	1.96±0.62	P=0.94
Preoperative Monocyte count(10^^9^/L)		0.43±0.15	0.42±0.14	P=0.71
Preoperative Hematocrit(L/L)		0.40±0.04	0.40±0.04	P=0.23
Preoperative Mean Corpuscular Volume(fL)		92.42±4.06	90.38±7.55	P=0.02
Preoperative Mean Corpuscular Hemoglobin(pg)		30.87±1.44	30.24±2.75	P=0.04
Preoperative Mean Corpuscular Hemoglobin Concentration(g/L)		334.08±12.55	334.78±13.56	P=0.57
Preoperative Red Cell Distribution Width(%)		12.48±0.64	12.70±1.25	P=0.09
Preoperative Platelet Distribution Width(fL)		12.32±1.95	12.85±1.57	P=0.68
Preoperative Hemoglobin Level(g/L)		134.05±14.01	137.06±51.42	P=0.54
Preoperative Platelet Count(10^^9^/L)		210.33±59.82	219.41±55.91	P=0.16
Preoperative Fibrinogen(g/L)		2.90±0.84	2.72±0.63	P=0.04
Preoperative Fibrin Degradation Products(mg/L)		2.71±0.59	2.58±0.42	P=0.10
Preoperative D-Dimer(mg/L)		0.63±0.77	0.39±0.38	P<0.01
Preoperative Albumin(g/L)		38.45±5.04	39.90±3.66	P<0.01
Preoperative Sodium Level(mmol/L)		141.84±2.10	141.46±1.98	P=0.12
Tumor Location	Left Right	75(41.9%) 100(58.1%)	367(44.0%) 471(56.0%)	P=0.58
Tumor Number	Single Multiple	126(72.1%) 49(27.9%)	630(75.1%) 208(24.9%)	P=0.65
Type	Adenocarcinoma Squamous Cell Carcinoma Others	150(86.0%) 19(3.5%) 6(10.5%)	734(87.7%) 79(2.9%) 25(9.4%)	P=0.61
Tumor Size(cm)		1.57±1.41	1.39±0.92	P=0.14
CEA Level(ng/mL)		2.15±1.59	2.78±16.86	P=0.74
NSE Level(ng/mL)		11.73±2.39	11.56±2.31	P=0.73

### 3.2 Feature selection

In the univariate analysis and Lasso regression analysis, variables that showed statistical significance included age, preoperative mean corpuscular volume (MCV), preoperative mean corpuscular hemoglobin (MCH), preoperative fibrinogen level, preoperative D-dimer level, and preoperative albumin level (**Table 2**). These six predictors were selected to construct the model.

**Table 2:** Univariate and Lasso Regression Analysis Results.

Variable	β	P-value
Age	0.0712	P<0.01
Preoperative Mean Corpuscular Volume (fL)	0.130	P=0.02
Preoperative Mean Corpuscular Hemoglobin (pg)	0.161	P=0.04
Preoperative Fibrinogen Level (g/L)	0.269	P=0.04
Preoperative D-Dimer Level (mg/L)	0.592	P<0.01
Preoperative Albumin Level (g/L)	-0.113	P<0.01

### 3.3 Model performance

**Figure 1:**
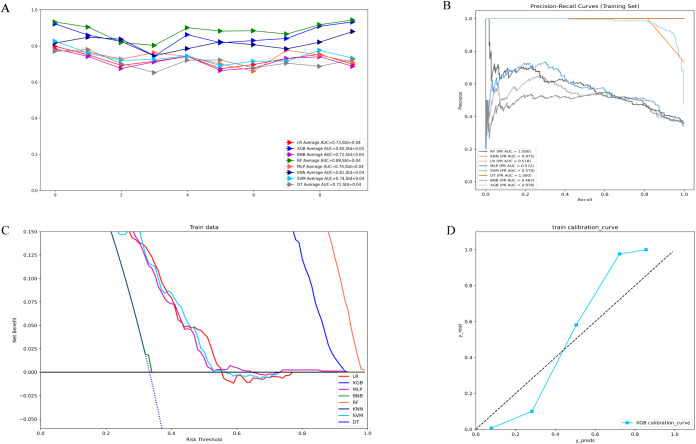
**(A)** Ten-fold cross-validation results of different machine models in the training set. **(B)** PR curves of different machine learning models in the training set. **(C)** DCA curves of different machine learning models in the training set. **(D)** Calibration curves of the best models in the training set. LR, logistic regression; XGB, extreme gradient boosting; BNB, Bernoulli Naïve Bayes; RF, random forest;MLP, multilayer perceptron;KNN, k-nearest neighbor;SVM, support vector machine; DT, decision tree.

**Figure 1A** illustrates the results of ten-fold cross-validation, demonstrating that the RF model achieved the highest performance with an average AUC of 0.88 (std = 0.04), outperforming XGB (AUC = 0.85, std = 0.05), LR (AUC = 0.73, std = 0.04), SVM (AUC = 0.74, std = 0.04), MLP (AUC = 0.73, std = 0.03), KNN (AUC = 0.81, std = 0.04), BNB (AUC = 0.72, std = 0.04), and DT (AUC = 0.71, std = 0.04). However, the RF model exhibited overfitting, as indicated by its AUPR in the training set. After excluding RF, the XGB model demonstrated the highest AUPR of 0.978 (**Figure 1B**). Additionally, XGB had the lowest Brier score among all models, measuring 0.0588. Based on training data, the DCA curve further confirmed the high reliability of XGB (**Figures 1C, D**).

**Figure 2:**
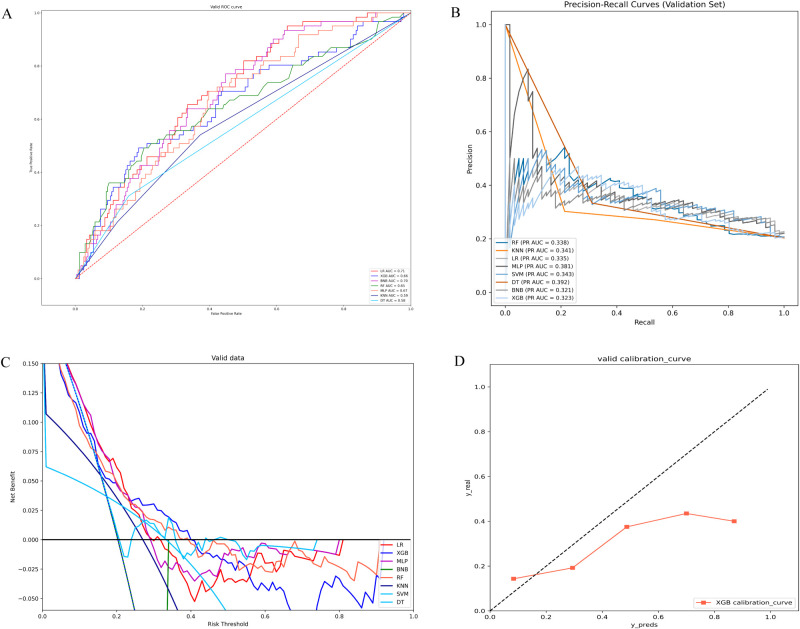
**(A)** ROC curves of different machine learning models in the validation set. **(B)** PR curves of different machine learning models in the validation set. **(C)** DCA curves of different machine learning models in the validation set. **(D)** Calibration curves of different machine learning models in the validation set; LR, logistic regression; XGB, extreme gradient boosting; BNB, Bernoulli Naïve Bayes; RF, random forest; MLP, multilayer perceptron;KNN, k-nearest neighbor; SVM, support vector machine; DT, decision tree.

**Figure 3:**
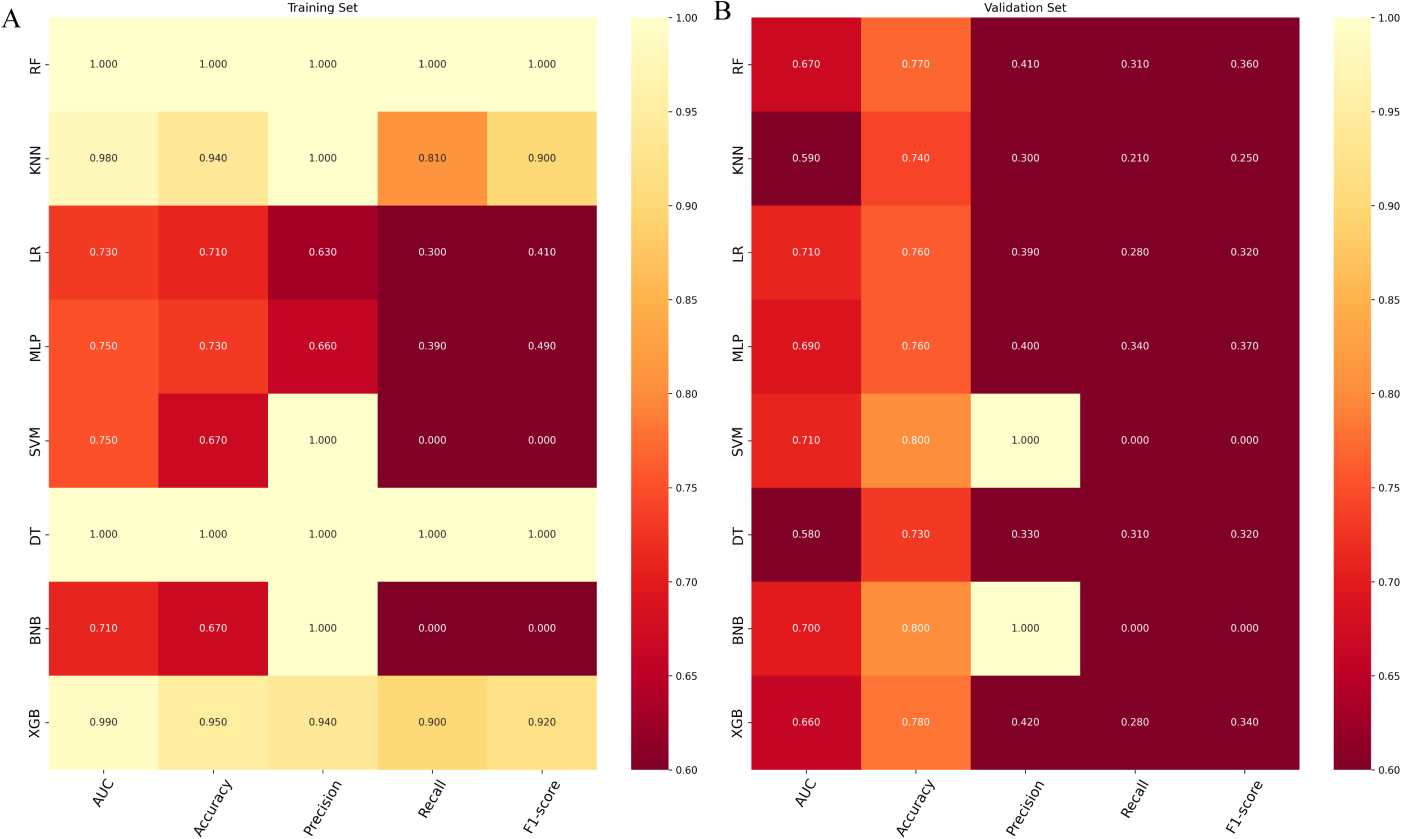
**(A)** Prediction performance of different models in the training set. **(B)** Prediction performance of different models in the validation set. RF, random forest; KMN, k-nearest neighbor; LR, logistic regression; MLP, multilayer perceptron; SVM, support vector machine; DT, decision tree; BNP, Bernoulli Naïve Bayes; XGB, extreme gradient boosting.

In the validation set, XGB achieved a notable AUC of 0.66, along with superior accuracy, precision, sensitivity, and F1 score (**Figures 2A**, **3B**). Moreover, it outperformed other models in terms of AUPR (**Figure 2B**). The XGB model also attained the highest MCC in the validation set, reaching 0.61. As shown in **Figure 3A**, XGB also demonstrated excellent prediction performance in the training set. Both DCA and clinical decision curves indicated that XGB had better clinical decision-making and predictive capabilities than the other seven models (**Figures 2C, D**). Given its robust predictive performance in the validation set, we designated XGB as the optimal model.

### 3.4 Importance of features in making predictions

**Figure 4:**
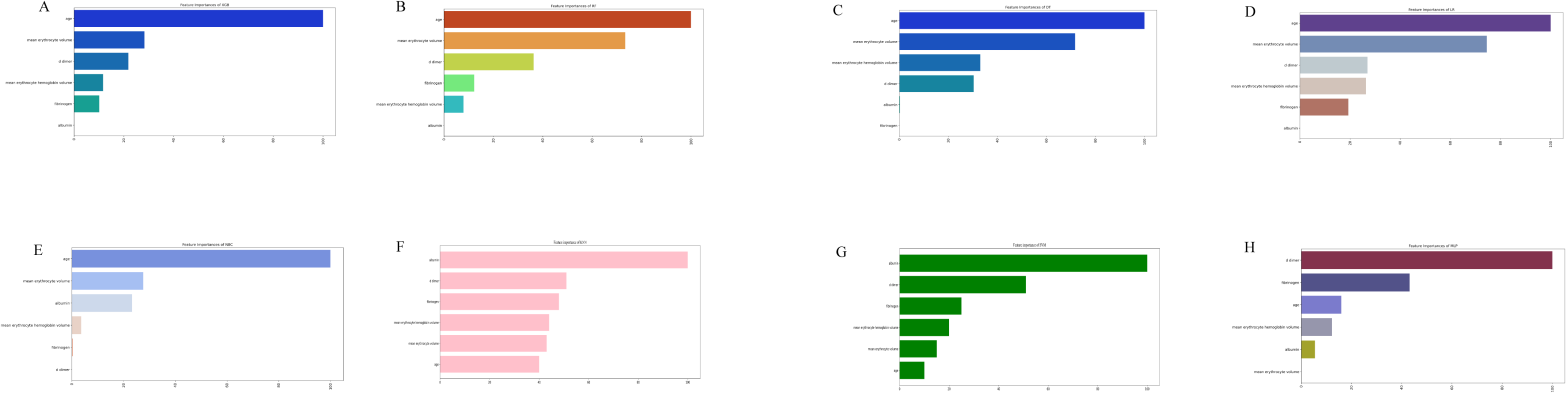
The importance of Variables in each prediction model. **(A)** Feature Importance of XGB. **(B)** Feature Importance of RF. **(C)** Feature Importance of DT. **(D)** Feature Importance of LR. **(E)** Feature Importance of NBC. **(F)** Feature Importance of KNN. **(G)** Feature Importance of SVM. **(H)** Feature Importance of MLP. LR, logistic regression; DT, decision tree; RF, random forest; XGB, extreme gradient boosting; NBC, Naïve Bayes Classifier; MLP, multilayer perceptron; SVM, support vector machine; KMN, k-nearest neighbor.

The significance of each feature in thrombosis prediction was evaluated using the importance ranking principle, with results shown in **Figure 4**. In most machine-learning models, age and mean erythrocyte volume were identified as the most influential predictors. Conversely, albumin consistently ranked as the least significant variable, though it still played a role in prediction. In the XGB model, features were ranked in descending order of importance as follows: age, mean erythrocyte volume, D-dimer, mean erythrocyte hemoglobin volume, fibrinogen, and albumin. Although individual models exhibited slight differences in ranking, the overall trend remained consistent.

### 3.5 Risk prediction of postoperative thrombosis in patients with lung cancer

**Figure 5:**
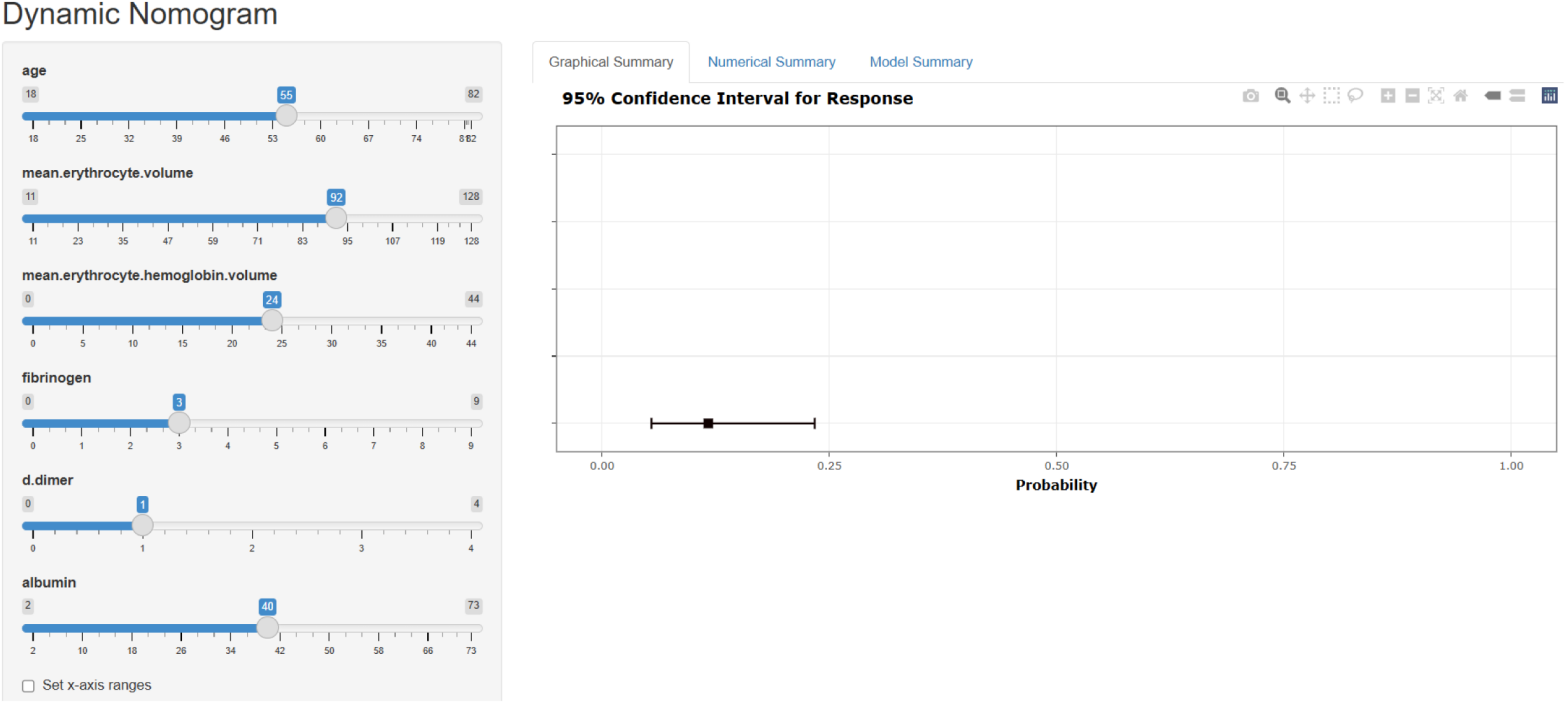
A web calculator for predicting the risk of thrombus in postoperative patients with lung cancer.

To enhance clinical utility, we developed an online prediction tool based on the XGB model to assess postoperative thrombosis risk in lung cancer patients. Although the XGB model exhibits strong predictive capabilities, its complexity limits direct application in clinical practice. This web-based calculator (https://cz2679994624.shinyapps.io/dynnomapp/) enables clinicians to input preoperative clinical and laboratory parameters to estimate an individual’s thrombosis risk after surgery. **Figure 5** provides a visual representation of the calculator interface.

## 4 Discussion

In this study, we developed and validated an XGBoost-based machine learning model to predict postoperative thrombosis risk in lung cancer patients. The model incorporates six key preoperative features: Age, Mean Corpuscular Volume, D-Dimer Level, Mean Corpuscular Hemoglobin, Fibrinogen Level and Albumin Level. The importance ranking of these variables aligns with previous research, reinforcing their predictive value in thrombosis risk assessment.

Several studies have explored the association between hypoalbuminemia and increased VTE risk. For example, Lionaki et al. (16) found that decreased albumin was an independent risk factor for VTE in patients with membranous nephropathy—a population already known to have a high baseline thrombotic risk. While this limits the generalizability of their findings, the proposed mechanisms remain biologically plausible and relevant across different populations. Albumin serves as a cofactor interacting with plasminogen, fibrin, and tissue plasminogen activators, thereby exerting anticoagulant effects by inhibiting fibrin polymerization and platelet aggregation (17, 18). It also enhances antithrombin activity, similar to the action of heparin (19). Importantly, this association has also been observed in broader populations. Folsom et al. (20) reported that lower serum albumin levels were associated with a higher risk of VTE in the general population, and Chi et al. (21) demonstrated an inverse relationship between albumin levels and VTE risk in acutely ill hospitalized patients. These studies suggest that hypoalbuminemia may reflect a prothrombotic state through its influence on coagulation and fibrinolysis pathways. When albumin levels fall below 35 g/L, the risk of thrombosis appears to increase, potentially due to elevated levels of fibrinogen and coagulation factors and impaired fibrinolytic activity (21).

Fibrinogen is a large and complex glycoprotein that is converted to fibrin in the coagulation cascade, forming a fibrin clot to stop bleeding (22). Studies have shown (23) that fibrinogen is an acute phase reactant produced by the liver during inflammation or ischemia, and its elevation is associated with an increased risk of VTE. The cause may be that surgery in lung cancer patients causes vascular trauma, activating internal coagulation factors and promoting a large release of thrombin, which converts soluble plasma fibrinogen into insoluble fibrin, leading to coagulation and venous thrombosis (24).

In the Khorana clinical prediction model, the Caprini clinical prediction model, and other studies (25, 26), older patients are considered to have a higher risk of VTE, with postoperative VTE risk doubling for every 10 years of age. This may be due to poor venous conditions in older patients, loss of muscle tone, and age-related degeneration, leading to increased venous stasis and endothelial damage. Older patients may also have venous diseases that increase outflow resistance, making thrombosis more likely (24, 27, 28). Studies (29) have shown that the postoperative VTE incidence in patients over 60 years of age is significantly higher than in those under 60.

Previous studies (25, 29) have confirmed that D-dimer is an independent risk factor for VTE formation, and higher preoperative D-dimer levels indicate a higher risk of VTE (30, 31). D-dimer is a specific molecular marker produced by the enzymatic breakdown of cross-linked fibrin by plasmin, and it reflects the degree of secondary fibrinolysis in the body. It is a sensitive indicator of fibrinolytic activity and coagulation status. Monitoring preoperative D-dimer levels helps clinicians identify patients at high risk for VTE or those in a pre-thrombosis state. Some studies suggest setting the D-dimer threshold for VTE risk assessment at 0.2 mg/L.

The characteristics of red blood cells are closely related to thrombosis, although there is limited research on the relationship between red blood cell volume and VTE formation. Some studies suggest that as the fibrin network contracts during thrombosis, red blood cells undergo morphological changes from their biconcave shape to various forms. When red blood cell volume increases, these cells have more difficulty changing shape, and their deformability decreases. Reduced deformability can result in insufficient clot contraction and microvascular occlusion, which may be a mechanism for VTE formation (32).

There is limited research on the relationship between changes in MCH and VTE. However, elevated MCH indicates increased blood viscosity and slower blood flow, which may activate platelets and fibrin, increasing the risk of venous stasis and thrombosis in the lower extremities.

To the best of our knowledge, this study represents the first instance of using machine learning algorithms and real-world data to predict the risk of postoperative thrombosis in lung cancer patients. A key strength of this research is the selection of six readily available variables to construct the model, enhancing its clinical applicability. Our predictive model enables clinicians to accurately assess the likelihood of postoperative thrombosis in lung cancer patients, allowing for preoperative optimization of anticoagulation strategies—such as adjusting low-molecular-weight heparin or anticoagulant regimens—for high-risk individuals. For patients who cannot tolerate anticoagulation, alternative preventive measures such as intermittent pneumatic compression (IPC) or graduated compression stockings (GCS) can be utilized. These approaches may help reduce the incidence of postoperative deep vein thrombosis (DVT) and pulmonary embolism (PE), ultimately improving postoperative safety.

Nevertheless, we acknowledge several limitations in our study. First, as a single-center retrospective study utilizing real-world data, there is a potential risk of bias. Second, our model was developed based on data from Chinese patients, and additional data from international populations are needed to refine the model and enhance its generalizability. Third, although we evaluated the model’s performance using an internal validation cohort, independent external validation from other medical centers or diverse populations is still required. Fourth, although tumor histological type was included in the univariate analysis and did not show a significant association with perioperative VTE, we note that the majority of patients (87.3%) had adenocarcinoma, which limited the feasibility of a meaningful histological subgroup analysis. In addition, tumor stage may influence laboratory parameters and VTE risk. However, due to incomplete staging data in this retrospective dataset, we were unable to include this factor in the current analysis. Future prospective studies with comprehensive staging data are warranted to further explore the relationship between tumor characteristics and thrombosis risk. Finally, another notable limitation pertains to the thromboprophylaxis strategy employed in our cohort. Postoperative patients did not receive routine prophylactic anticoagulation. Instead, a reactive approach was adopted, where low-molecular-weight heparin (LMWH) was initiated only after ultrasound-confirmed thrombosis. This strategy may have contributed to the observed VTE incidence and complicates comparisons with studies that employed standardized prophylactic anticoagulation protocols.

## 5 Conclusion

In conclusion, we developed and validated a novel model using machine learning algorithms, which incorporates six commonly used clinical variables and demonstrates overall good performance, with an AUROC of 0.66, an AUPRC of 0.323, an accuracy of 0.78, a precision of 0.49. This model effectively identifies high-risk individuals and aids in preventing the development of perioperative venous thromboembolism (VTE) in lung cancer patients.

## Data Availability

The original contributions presented in the study are included in the article/supplementary material. Further inquiries can be directed to the corresponding authors.

## References

[1] Siegel RL Giaquinto AN Jemal A. Cancer statistics. CA Cancer J Clin. (2024) 74:12–49. doi: 10.3322/caac.21820 38230766

[2] Crosby D Bhatia S Brindle KM Coussens LM Dive C Emberton M. Early detection of cancer. Science. (2022) 375:eaay9040. doi: 10.1126/science.aay9040 35298272

[3] Gordon RJ Lombard FW. Perioperative venous thromboembolism: A review. Anesth Analg. (2017) 125:403–12. doi: 10.1213/ane.0000000000002183 28640782

[4] Zhang Y Yang Y Chen W Guo L Liang L Zhai Z. Prevalence and associations of VTE in patients with newly diagnosed lung cancer. Chest. (2014) 146:650–8. doi: 10.1378/chest.13-2379 24676401

[5] Pastori D Cormaci VM Marucci S Franchino G Del Sole F Capozza A. A comprehensive review of risk factors for venous thromboembolism: from epidemiology to pathophysiology. Int J Mol Sci. (2023) 24:14–24. doi: 10.3390/ijms24043169 PMC9964264 36834580

[6] Spyropoulos AC Hussein M Lin J Battleman D. Rates of venous thromboembolism occurrence in medical patients among the insured population. Thromb Haemost. (2009) 102:951–7. doi: 10.1160/th09-02-0073 19888534

[7] Thrall JH Li X Li Q Cruz C Do S Dreyer K. Artificial intelligence and machine learning in radiology: opportunities, challenges, pitfalls, and criteria for success. J Am Coll Radiol. (2018) 15:504–8. doi: 10.1016/j.jacr.2017.12.026 29402533

[8] Deo RC. Machine learning in medicine. Circulation. (2015) 132:1920–30. doi: 10.1161/circulationaha.115.001593 PMC5831252 26572668

[9] Shouval R Bondi O Mishan H Shimoni A Unger R Nagler A. Application of machine learning algorithms for clinical predictive modeling: a data-mining approach in SCT. Bone Marrow Transplant. (2014) 49:332–7. doi: 10.1038/bmt.2013.146 24096823

[10] Jiang T Gradus JL Rosellini AJ. Supervised machine learning: A brief primer. Behav Ther. (2020) 51:675–87. doi: 10.1016/j.beth.2020.05.002 PMC7431677 32800297

[11] Xu Y Hosny A Zeleznik R Parmar C Coroller T Franco I. Deep learning predicts lung cancer treatment response from serial medical imaging. Clin Cancer Res. (2019) 25:3266–75. doi: 10.1158/1078-0432.Ccr-18-2495 PMC6548658 31010833

[12] Placido D Yuan B Hjaltelin JX Zheng C Haue AD Chmura PJ. A deep learning algorithm to predict risk of pancreatic cancer from disease trajectories. Nat Med. (2023) 29:1113–22. doi: 10.1038/s41591-023-02332-5 PMC10202814 37156936

[13] Maabreh RSA Alazzam MB AlGhamdi AS. Machine learning algorithms for prediction of survival curves in breast cancer patients. Appl Bionics Biomech. (2021) 2021:9338091. doi: 10.1155/2021/9338091 34845416 PMC8627349

[14] Wiberg H Yu P Montanaro P Mather J Birz S Schneider M. Prediction of neutropenic events in chemotherapy patients: A machine learning approach. JCO Clin Cancer Inform. (2021) 5:904–11. doi: 10.1200/cci.21.00046 34464160

[15] Zhou Y Hou Y Hussain M Brown SA Budd T Tang WHW. Machine learning-based risk assessment for cancer therapy-related cardiac dysfunction in 4300 longitudinal oncology patients. J Am Heart Assoc. (2020) 9:e019628. doi: 10.1161/jaha.120.019628 33241727 PMC7763760

[16] Lionaki S Derebail VK Hogan SL Barbour S Lee T Hladunewich M. Venous thromboembolism in patients with membranous nephropathy. Clin J Am Soc Nephrol. (2012) 7:43–51. doi: 10.2215/cjn.04250511 22076873 PMC3265338

[17] Mahmoodi BK Gansevoort RT Veeger NJ Matthews AG Navis G Hillege HL. Microalbuminuria and risk of venous thromboembolism. Jama. (2009) 301:1790–7. doi: 10.1001/jama.2009.565 19417196

[18] Tang X Zhang Z Fang M Han Y Wang G Wang S. Transferrin plays a central role in coagulation balance by interacting with clotting factors. Cell Res. (2020) 30:119–32. doi: 10.1038/s41422-019-0260-6 PMC7015052 31811276

[19] Jain V Ploutz-Snyder R Young M Charvat JM Wotring VE. Potential venous thromboembolism risk in female astronauts. Aerosp Med Hum Perform. (2020) 91:432–9. doi: 10.3357/amhp.5458.2020 32327017

[20] Folsom AR Lutsey PL Heckbert SR Cushman M. Serum albumin and risk of venous thromboembolism. Thromb Haemost. (2010) 104:100–4. doi: 10.1160/th09-12-0856 PMC2902783 20390234

[21] Chi G Gibson CM Liu Y Hernandez AF Hull RD Cohen AT. Inverse relationship of serum albumin to the risk of venous thromboembolism among acutely ill hospitalized patients: Analysis from the APEX trial. Am J Hematol. (2019) 94:21–8. doi: 10.1002/ajh.25296 30252149

[22] Weisel JW. Fibrinogen and fibrin. Adv Protein Chem. (2005) 70:247–99. doi: 10.1016/s0065-3233(05)70008-5 15837518

[23] Kara H Bayir A Degirmenci S Kayis SA Akinci M Ak A. D-dimer and D-dimer/fibrinogen ratio in predicting pulmonary embolism in patients evaluated in a hospital emergency department. Acta Clin Belg. (2014) 69:240–5. doi: 10.1179/2295333714y.0000000029 25012747

[24] De Stefano V Za T Rossi E. Venous thromboembolism in multiple myeloma. Semin Thromb Hemost. (2014) 40:338–47. doi: 10.1055/s-0034-1370793 24599441

[25] Cui S Chen S Li H Ke L Liu Y Jiang R. Risk factors for venous thromboembolism and evaluation of the modified Caprini score in patients undergoing lung resection. J Thorac Dis. (2020) 12:4805–16. doi: 10.21037/jtd-20-1279 PMC7578470 33145053

[26] Di W Xu H Xue T Ling C. Advances in the prediction and risk assessment of lung cancer-associated venous thromboembolism. Cancer Manag Res. (2021) 13:8317–27. doi: 10.2147/cmar.S328918 PMC8575248 34764694

[27] Vollans S Chaturvedi A Sivasankaran K Madhu T Hadland Y Allgar V. Symptomatic venous thromboembolism following circular frame treatment for tibial fractures. Injury. (2015) 46:1108–11. doi: 10.1016/j.injury.2015.04.003 25910819

[28] Rapp CM Shields EJ Wiater BP Wiater JM. Venous thromboembolism after shoulder arthoplasty and arthroscopy. J Am Acad Orthop Surg. (2019) 27:265–74. doi: 10.5435/jaaos-d-17-00763 30480588

[29] Wang P Zhao H Zhao Q Ren F Shi R Liu X. Risk factors and clinical significance of D-dimer in the development of postoperative venous thrombosis in patients with lung tumor. Cancer Manag Res. (2020) 12:5169–79. doi: 10.2147/cmar.S256484 PMC7335272 32636679

[30] Tian B Song C Li H Zhang W Chen Q Chen S. The significance of perioperative coagulation and fibrinolysis related parameters after lung surgery for predicting venous thromboembolism: a prospective, single center study. J Thorac Dis. (2018) 10:2223–30. doi: 10.21037/jtd.2018.03.174 PMC5949486 29850126

[31] Li J Qiang WM Wang Y Wang XY. Development and validation of a risk assessment nomogram for venous thromboembolism associated with hospitalized postoperative Chinese breast cancer patients. J Adv Nurs. (2021) 77:473–83. doi: 10.1111/jan.14571 33159325

[32] Evtugina NG Peshkova AD Pichugin AA Weisel JW Litvinov RI. Impaired contraction of blood clots precedes and predicts postoperative venous thromboembolism. Sci Rep. (2020) 10:18261. doi: 10.1038/s41598-020-75234-y 33106547 PMC7589563

